# Lichen sclerosus and the association with subsequent psychiatric disorders

**DOI:** 10.3389/fmed.2025.1653347

**Published:** 2025-10-21

**Authors:** Salam Alfarsi, Andreas Recke, Katja Bieber, Diamant Thaçi, Ralf J. Ludwig, Philip Curman

**Affiliations:** 1 Lübeck Institute of Experimental Dermatology, University of Lübeck, Lübeck, Germany; 2 Department of Dermatology, University-Hospital Schleswig-Holstein (UKSH), Lübeck, Germany; 3 Institute and Comprehensive Centre for Inflammation Medicine, University-Hospital Schleswig-Holstein, Lübeck, Germany; 4 Dermato-Venereology Clinic, Karolinska University Hospital, Stockholm, Sweden; 5 Division of Dermatology and Venereology, Department of Medicine (Solna), Karolinska Institutet, Stockholm, Sweden; 6 Department of Medical Epidemiology and Biostatistics, Karolinska Institutet, Stockholm, Sweden

**Keywords:** psychiatric disease, Lichen sclerosus, TriNetX, cohort study, mental health, depression

## Abstract

**Background:**

Lichen sclerosus (LS) is an acquired, non-communicable, chronic inflammatory disease that predominantly affects the genital area and may lead to substantial impairment in quality of life. While some studies reported elevated rates of depression and anxiety among patients with LS, the available evidence is limited by often small sample sizes, cross-sectional designs, narrow matching, and limited consideration of sex- or race-disparities. Moreover, the risk of a broader spectrum of psychiatric disorders remains insufficiently characterized.

**Objective:**

To evaluate the risk of a larger spectrum of psychiatric disorders following a diagnosis of LS in a retrospective cohort study.

**Methods:**

The US Collaborative Network of TriNetX was used to create a propensity-score-matched cohort of individuals with LS and non-LS controls (*n* = 42,581 per cohort). Risk of psychiatric disorders following the index events was analyzed in a retrospective cohort study. Several sensitivity analyses were conducted to assess robustness of the findings. Subgroup analyses were performed to identify potential sex- or racial-disparities.

**Results:**

Within 5 years, 3.92% of patients with LS as opposed to 3.43% of controls were subsequently diagnosed with a depressive episode (HR 1.31, CI 1.22–1.40, *p* < 0.0001). Furthermore, risks of recurrent major depression (HR 1.71, CI 1.48–1.98, *p* < 0.0001) and reaction to severe stress (HR 1.62, CI 1.45–1.80, *p* < 0.0001) were increased in patients with LS. These risks seemed more pronounced in those of White ethnicity and in women. Risks for suicidal ideations, suicide attempts, and schizophrenia were not different between patients and matched controls.

**Conclusion:**

Patients with LS are at a moderately increased risk of depression and stress-related psychiatric disorders.

## 1 Introduction

Lichen sclerosus (LS) is an acquired, non-communicable, chronic inflammatory disease. The disease most commonly affects the anogenital region, though extragenital involvement can occur in a minority of cases. The disease predominantly affects postmenopausal women but can also manifest in men and at younger age (1). Clinically, LS is characterized by porcelain-white, atrophic plaques, often accompanied by pruritus, pain, and, in chronic cases, architectural changes and scarring of the vulva, perineum, or penis. Complications include sexual and urinary dysfunction, significant impairment in quality of life, and an increased risk of squamous cell carcinoma, particularly in the vulva and penis. The diagnosis of LS is based on characteristic clinical features and supported by histopathology where needed (epidermal atrophy, homogenization of collagen in the upper dermis, thickened basement membrane, and a band-like lymphocytic infiltrate). (1, 2).

In general, there is a well-documented inverse correlation between mental health and sexual dysfunction (3, 4). In line, some studies suggest a higher prevalence of depressive symptoms, anxiety, and reduced quality of life in patients with LS compared to controls, although most are limited by sample size, cross-sectional design, and lack matching accounting for risk factors of psychiatric disease (5). In more detail, in 765 women with LS 42% were also diagnosed with a depression as opposed to 22% of the controls, amounting to an odds ratio (OR) of 2.16 (95% confidence interval [CI] 1.82–2.57). Likewise, an increased odds of anxiety was also noted in this study (6). A Swedish population-based study investigating comorbidities associated with LS included 154,424 patients and a sex- and age-matched control group of 463,273 individuals without LS. Regarding mental health outcomes, the study documented significant associations of LS with alcohol abuse, and nicotine dependence (2). Furthermore, among women diagnosed with LS, those with vulvar involvement showed an increased risk of receiving prescriptions for antidepressants and benzodiazepines, as well as being diagnosed with depression or anxiety disorders, compared to women with LS without vulvar involvement (7). A systematic review of several smaller studies examined the prevalence of depression in patients with vulvar inflammatory dermatoses, including LS. The study reported depression prevalence estimates ranging from 14 to 50%, depending on assessment tools and study populations. However, only eight studies employed validated depression instruments, and most of the studies were cross-sectional or retrospective chart reviews assessing associations rather than incident risk. Thus, the temporal relationship between LS diagnosis and subsequent psychiatric disease was rarely addressed (8). Another limitation of these studies is reverse causation, as individuals with psychiatric disorders may have increased healthcare utilization, thereby raising the likelihood of receiving an LS diagnosis.

Overall, this underscores the need for longitudinal studies assessing the risk of psychiatric disorders following LS diagnosis. Thus, to clarify the potential impact of LS on mental health, we conducted a large-scale retrospective cohort study investigating a spectrum of psychiatric diseases. To reduce confounding, we employed propensity-score matching. To strengthen the temporal relationship, we assessed psychiatric outcomes only after the LS diagnosis.

## 2 Materials and methods

### 2.1 Study design and data source

**Figure 1:**
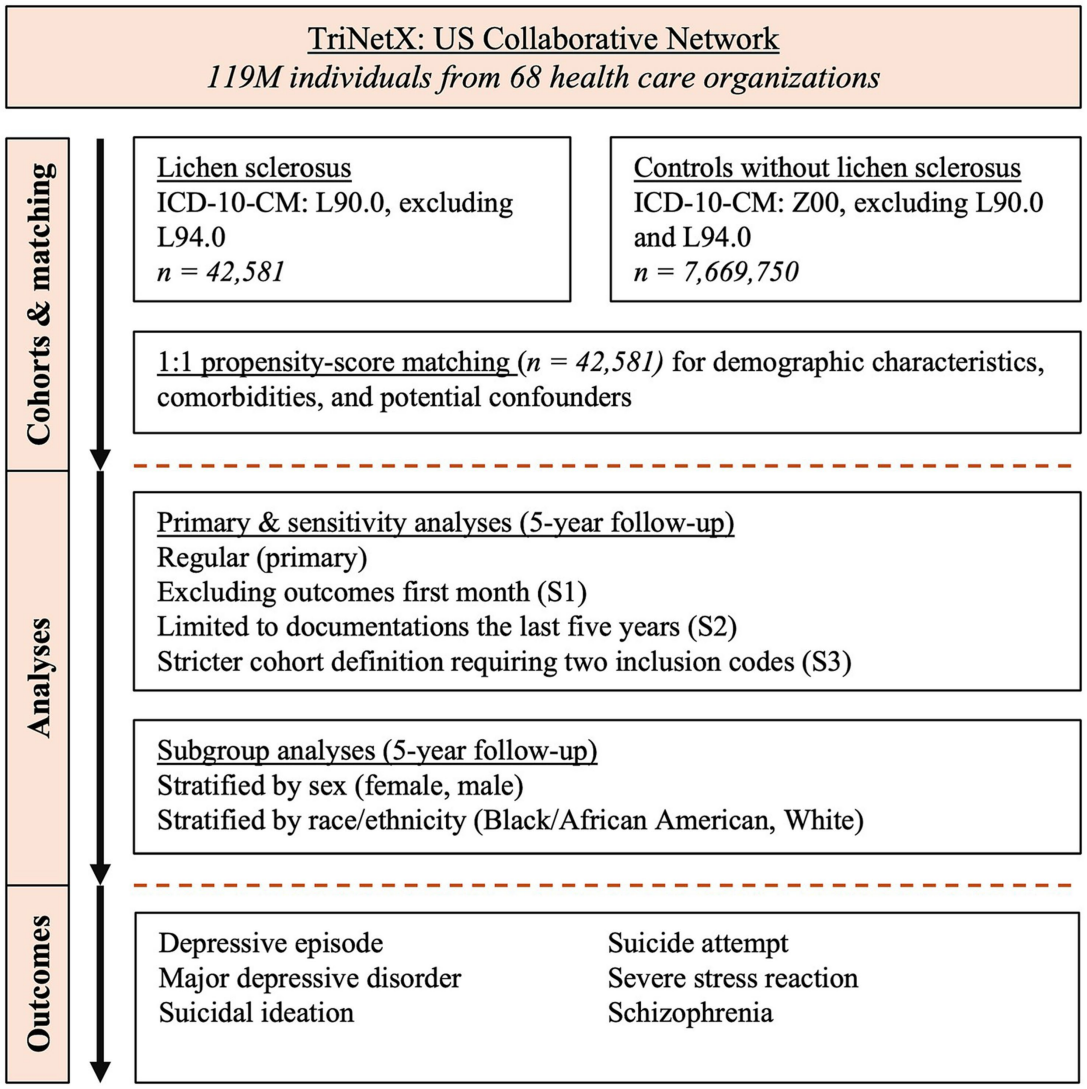
Study flow chart.

We conducted a retrospective cohort study using data from the US Collaborative Network of TriNetX (Figure 1). The TriNetX electronic health records (EHR) database was utilized following established protocols (9–12). This database was chosen for its extensive collection of EHRs and comprehensive documentation of covariates (13). Through a collaboration with TriNetX, researchers from UKSH gained access to the database. We identified EHRs for patients diagnosed with LS and a propensity-score matched (PSM) non-LS control group. The risks of six psychiatric disorders, namely: suicidal ideations, suicide attempts, depressive episodes, major depressive disorder, schizophrenia, and stress-related disorders, were compared between LS patients and controls. PSM was applied to enhance comparability between groups with respect to demographic characteristics, comorbidities, and potential confounders, as detailed in Table 1. Additionally, three sensitivity analyses were conducted to assess the robustness of the results. Analyses stratified by sex and race were performed to examine potential disparities. Study endpoints were defined using International Classification of Diseases, 10^th^ edition, Clinical Modification (ICD-10-CM) codes before data collection.

**Table 1:** Baseline characteristics before and after propensity-score matching in lichen sclerosus (cases) and controls for the primary and S1 analyses.

Characteristic	Before matching			After matching		
	Lichen sclerosus	Controls	Std. diff.	Lichen sclerosus	Controls	Std. diff.
Number of participants	42,581	7,669,750		42,581	42,581	
Follow-up (days) median (interquartile range)	1,010 (1,425)	1,323 (1,356)		1,010 (1,425)	1,362 (1,303)	
Age at index (years, SD)	60.8 ± 14.1	47 ± 18.1	0.8466	60.8 ± 14.1	60.8 ± 14.1	0.0004
Female (%)	93.5	51.6	1.0619	93.5	93.5	0.0006
White (%)	81.1	63.9	0.3919	81.1	81.2	0.0029
Diseases of the circulatory system (I00–I99, %)	43.8	29.3	0.3045	43.8	43.8	0.0002
Diseases of the respiratory system (J00–J99, %)	37.8	29.6	0.1729	37.8	37.7	0.0013
Neoplasms (C00–D49, %)	35.6	14.9	0.4893	35.6	35.4	0.0030
Diseases of the blood and blood-forming organs and certain disorders involving the immune mechanism (D50–D89, %)	17.1	10.3	0.1969	17.1	17.1	0.0007
Diseases of the musculoskeletal system and connective tissue (M00–M99, %)	58.0	37.4	0.4212	58.0	57.9	0.0020
Endocrine, nutritional and metabolic diseases (E00–E89, %)	55.5	35.5	0.4111	55.5	55.5	0.0005
Diseases of the digestive system (K00–K95, %)	44.9	26.2	0.3987	44.9	44.9	0.0008
Overweight and obesity (E66, %)	16.1	9.8	0.1880	16.1	16.1	0.0003
Persons with potential health hazards related to socioeconomic and psychosocial circumstances (Z55–Z65, %)	1.1	1.1	0.0013	1.1	1.1	0.0068

SD, standard deviation; Std. diff., standardized difference.

### 2.2 Study population

The study was conducted from November to December 2024, with data collected from the US Collaborative Network of TriNetX. At the time of analysis, the database provided access to EHRs from over 119 million patients across 68 healthcare organizations (HCOs) in the United States. Patients diagnosed with LS were defined by an instance of ICD-10-CM: L90.0, excluding any code of L94.0 (localized scleroderma). Non-LS controls were defined by documentation of a healthcare encounter for general examination without complaint, suspected, or reported diagnosis (Z00), excluding those with codes for L94.0 or L90.0. To ensure sufficient data for PSM, a prior medical visit (at least 6 months before the respective index events) was mandated for all groups. The study included adult patients aged 18 years or older.

### 2.3 Outcomes

Outcomes were defined by the ICD-10-CM codes: Suicidal ideations (R45.851), suicide attempt (T14.91), recurrent major depressive disorder (MDD, F33), depressive episode (F32), schizophrenia (F20), and reaction to severe stress, and adjustment disorders (F43). Any outcomes occurring before the index event were excluded at data retrieval.

### 2.4 Primary, sensitivity and subgroup analyses

In the primary analysis, outcomes were evaluated starting from 1 day after the index event and continuing up to 5 years post-index. For sensitivity analysis S1, to exclude outcomes that may have been present but not diagnosed or recorded at the time of the index event, only outcomes occurring from 1 month after the index event to 5 years later were included. In sensitivity analysis S2, to address potential bias due to changes in clinical practices, only electronic health records (EHRs) documented within the past 5 years were considered. In sensitivity analysis S3, a stricter case definition of LS was applied, requiring at least two separate documentations of L90.0. To mirror this approach in the control group, inclusion required at least two separate documentations of Z00 for S3. Outcomes occurring from 1 day to 5 years post-index were included in this analysis.

### 2.5 Sex and ethnicity analyses

For sex- and ethnicity-stratified analyses, the definitions used in the primary analysis were maintained, but data were specifically stratified by sex (female or male) and self-reported ancestry (Black or African American, or White).

### 2.6 Statistical analysis

A propensity-score for each patient was generated by logistic regression analysis (with exposure as the dependent variable) using the Python package scikit-learn. Matching was performed 1:1 using the greedy nearest neighbor approach with a cut-off distance of 0.1 pooled standard deviations of the logit of the propensity-score. Baseline characteristics were re-evaluated and reported after matching, differences were compared by *t*-test for continuous and *z*-test for binary or categorical variables. Relative risks and risk difference (RD) were calculated. Survival analysis was performed using the Kaplan–Meier method (KM) in Survival package v3.2-3 in R (R Foundation for Statistical Computing, Vienna, Austria) and validated by comparison with the outputs of SAS version 9.4 (SAS, Cary, NC). The proportionality assumption was tested by the coxph function in R’s Survival package. KM-curves were compared using the Log-rank test. A univariate Cox proportional hazards regression was used to express hazard ratios (HRs) with 95%-confidence intervals (CIs). Outcomes prior to index were excluded. Bonferroni correction was used to counter the bias of multiple testing (*α*_adj._ = 0.004).

### 2.7 Use of artificial intelligence

ChatGPT-4o (OpenAI LCC, San Francisco, California, USA) was used to assist in extracting data from tables and revising sections of the manuscript. All extracted data and revisions were thoroughly reviewed and validated by the authors. The authors take full responsibility for the accuracy, integrity, and final content of the manuscript.

## 3 Results

### 3.1 Baseline characteristics

Before PSM, the 42,581 LS cases and 7,669,750 non-LS controls differed markedly in age, sex, and comorbidities (e.g., neoplasms: 35.6% vs. 14.9%; circulatory diseases: 43.8% vs. 29.3%). After PSM, cohorts were balanced at 42,581 per group, with minimal differences in age (std. diff. = 0.0004), sex (std. diff. < 0.0006), and major comorbidities. Full cohort characteristics and matching results for the primary and S1 analysis are shown in Table 1. Cohort descriptions pertaining to S2-3 and subgroup analysis are shown in Supplementary Table 1.

### 3.2 LS is associated with increased risks for depressive episodes, MDD, and reaction to severe stress

**Figure 2:**
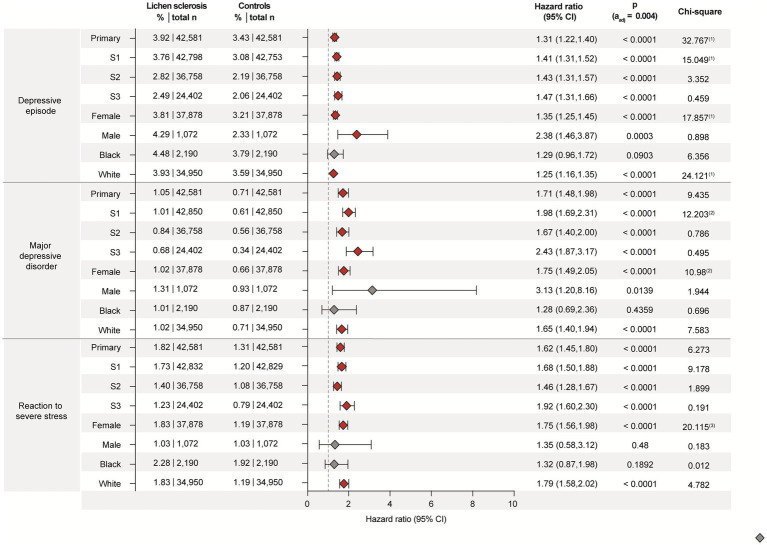
Lichen sclerosus (LS) is associated with increased risks for depression, major depressive disorder, and reaction to severe stress. Data were retrieved and analyzed using the US Collaborative Network of TriNetX. The figure displays hazard ratios (HRs) and 95% confidence intervals (CIs) for depressive episode, major depressive disorder, and reaction to severe stress in patients with LS vs. propensity-score matched non-LS controls. Risks are shown for the primary analysis, all three sensitivity analyses (S1–S3), and subgroups stratified by sex (male, female) and self-reported ethnicity (White, Black or African American). Chi-square values indicate proportionality of outcome distribution across groups. Please note that proportional hazards assumption was violated in some subgroup analyses, indicating that hazard ratios may vary over time and should be interpreted with caution.

We here document a significantly increased risk of being diagnosed with a depressive episode in patients with LS compared to non-LS controls (Figure 2). In more detail, 3.92% of LS patients received a diagnosis of depressive episode within 5 years of LS diagnosis, compared to 3.43% of controls during the same period (HR 1.305, CI 1.217–1.40, *p* < 0.0001). This elevated risk persisted across all three sensitivity analyses, with the strictest definition of LS (S3) demonstrating the highest risk (HR 1.47, CI 1.31–1.66, *p* < 0.0001). These findings suggest that the burden of depressive disorders in LS patients may increase as diagnostic criteria become more stringent, indicating a robust association. In the subgroup analyses stratified for sex or ethnicity, the risk for depressive episodes was observed in both females (HR 1.35, CI 1.25–1.45, *p* < 0.0001) and males (HR 2.38, CI 1.46–3.87, *p* < 0.0003). When stratified for self-reported ethnicity, an increased risk was observed in White (HR 1.25, CI 1.16–1.35, *p* < 0.0001), but not Black or African American patients with LS (HR 1.29, CI 0.96–1.72, *p* = 0.0903). Given the relatively low sample size of male (*n* = 1,072), or Black or African American (*n* = 2,190) patients with LS (Supplementary Table 1), subgroup analysis relating to male sex or Black or African American ancestry findings should be interpreted with caution.

Similar observations were made for MDD that was diagnosed in 1.05% of LS cases as opposed to 0.71% of non-LS controls during the 5-year follow-up (HR 1.71, CI 1.48–1.98, *p* < 0.0001, Figure 2). These results persisted across all three sensitivity analyses and showed the highest risk when the definition of cases was most stringent (HR 2.43, CI 1.87–3.17, *p* < 0.0001). In the sex-stratified analysis, the increased risk for MDD was observed for females (HR 1.75, CI 1.56–1.98, *p* < 0.0001, *n* = 37,878), but not males (HR 3.13, CI 1.20–8.16, *p* = 0.0139 [*α*_adj._ = 0.004], *n* = 1,072). As observed for depressive episodes, the risk of MDD following a diagnosis of LS was only observed in White, but not Black or African American patients with LS (Figure 2, Supplementary Table 1). Again, note that the relatively small sample size of Black or African American patients with LS limits statistical power.

Stress-related disorders, specifically reaction to severe stress, are also more frequently observed in patients with LS (1.82%) compared to non-LS controls (1.31%), translating into an HR of 1.62 (CI 1.45–1.80, *p* < 0.001, Figure 2). These results of the primary analysis were consistent in all three sensitivity analyses—again with the highest risk observed when the more stringent definition of cases was applied. In the subgroup analyses, female and White patients with LS had an elevated risk for a subsequent diagnosis of reaction to severe stress. This was not observed in male and Black or African American LS patients, which may again be due to the relatively small sample size of these two subgroups.

### 3.3 LS is not associated with increased risks for suicidal ideations, suicide attempts or schizophrenia

**Figure 3:**
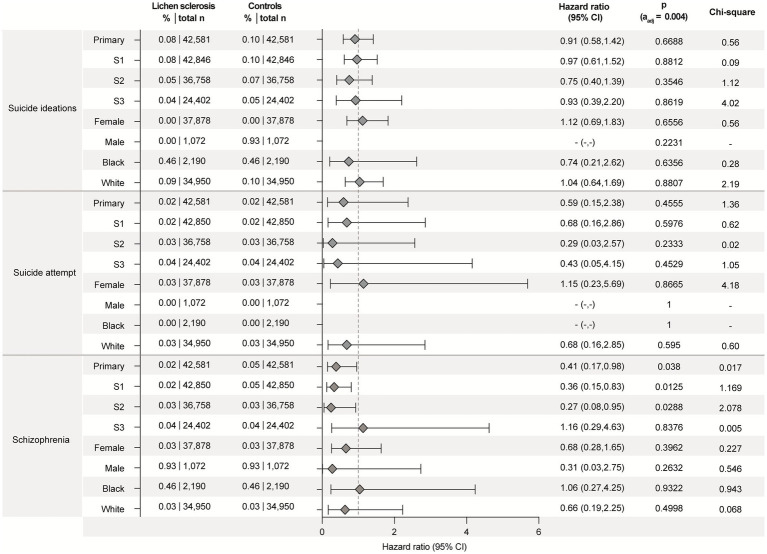
Lichen sclerosus (LS) is not associated with increased risks for suicidal ideation, suicide attempts, or schizophrenia. Data were retrieved and analyzed using the US Collaborative Network of TriNetX. The figure displays hazard ratios (HRs) and 95% confidence intervals (CIs) for suicidal ideation, suicide attempts, or schizophrenia in patients with LS vs. propensity-score matched non-LS controls. Risks are shown for the primary analysis, all three sensitivity analyses (S1–S3), and subgroups stratified by sex (male, female) and self-reported ethnicity (White, Black or African American). Chi-square values indicate proportionality of outcome distribution across groups. Please note that proportional hazards assumption was violated in some subgroup analyses, indicating that hazard ratios may vary over time and should be interpreted with caution.

No significant differences in the risk of suicidal ideation, suicide attempts, or schizophrenia were observed between LS patients and controls. These findings remained consistent across all sensitivity and subgroup analyses (Figure 3). However, the number of observed outcomes for each of these endpoints was low, which limits statistical power and should be considered when interpreting the results.

## 4 Discussion

In this large, retrospective cohort study using EHRs from over 42,000 patients diagnosed with LS, we found a significantly increased risk of being diagnosed with depressive episodes, MDD, and stress-related disorders, particularly reaction to severe stress, following LS diagnosis. These findings were robust across three sensitivity analyses and persisted in most subgroup analyses stratified by sex and self-reported ethnicity.

Our results extend upon previous insights from a small number of earlier, mostly cross-sectional studies suggesting an association between LS and psychiatric morbidity (2, 6, 8). Importantly, our study is among the first to analyze incident risk of psychiatric disorders after LS diagnosis using a temporal design, which allows for a better inference about directionality. The observed association is biologically and psychosocially plausible: LS frequently affects intimate body areas, often in a disfiguring and painful manner, contributing to sexual dysfunction, stigmatization, and psychological distress (1, 14, 15). The consistent increase in psychiatric risk across all sensitivity and most subgroup analyses suggests that mental health is an important but still underrecognized aspect of LS care.

While both female and male patients with LS showed elevated risks for depressive episodes, results for MDD were statistically robust only in females, possibly due to limited statistical power in the smaller male subgroup. Similarly, increased risk for depressive and stress-related disorders was observed in White patients, but not in Black or African American patients with LS. This might reflect genuine differences in disease perception, healthcare access, or diagnostic bias (16). However, the relatively small number of LS cases among Black or African American individuals diagnosed with LS is the most likely explanation for this finding. Notably, the HR for depressive episodes in Black or African American LS patients (HR 1.287, CI 0.96–1.724, *p* = 0.0903) pointed in the same direction as in White patients (HR 1.25, CI 1.157–1.349, *p* < 0.0001), suggesting a similar underlying effect that may have gone undetected due to limited power. Similar considerations apply to MDD and stress-related disorders, as well as for MDD and stress-related disorders in male patients with LS.

Notably, we found no evidence of increased risk for suicidal ideation, suicide attempts, or schizophrenia in patients with LS compared to controls. These findings were consistent across all sensitivity and subgroup analyses. However, low event counts limit statistical power and preclude definitive conclusions. In principle, this limitation could be addressed through a meta-analysis of existing studies. However, the literature on this topic is sparse at best: Only one study reported suicidal ideations in 6 out of 422 patients (1.4%) with vulvar LS (17). This figure is substantially higher than the 0.08% prevalence of suicidal ideation documented in our study. The apparent discrepancy likely reflects differences in study design: Prospective assessment with direct inquiry about suicidal thoughts (17) versus our retrospective cohort approach based on coded diagnoses. Thus, the prevalence of psychiatric disease is potentially underreported in LS.

Several limitations should be acknowledged. First, although propensity-score matching was used to minimize baseline imbalances, residual confounding from unmeasured variables (e.g., symptom severity, or access to mental health care) cannot be ruled out. Second, LS diagnosis was based on ICD-10-CM codes, which may vary in accuracy across healthcare providers and institutions. Third, psychiatric outcomes were also defined by diagnostic codes, which may not capture subclinical disease or undiagnosed cases. Fourth, although the temporal sequence of diagnoses can be established, the retrospective design does not permit causal inference, and residual or bidirectional confounding cannot be ruled out. Moreover, although outcomes were analyzed only after the LS diagnosis, diagnosis dates in EHRs may lag true clinical onset; therefore, our findings concern hazards of recorded diagnoses rather than definitive disease onset. Fifth, the relatively small number of male and Black or African American patients with LS limits the power of subgroup analyses and the generalizability of these findings to underrepresented populations. Sixth, in some cases, the proportional hazards assumption was violated. Yet, the chi-square and *p*-values calculated from the Schoenfeld residuals are sensitive to differences in time value transformations used by different software packages. For example, KM time transformations used by Survival and SAS software can yield different chi-square statistics, despite agreement in hazard ratio estimates. This discrepancy arises from the time shifts between transformed values. Thus, TriNetX advises to interpret these chi-square and *p*-values as qualitative guides to the degree of time variance in hazard ratios, rather than as exact quantitative metrics (9). Finally, outcome frequency for suicide-related endpoints and schizophrenia was low, precluding definite conclusions for these disorders.

In conclusion, our findings show that LS is associated with a modest but statistically and clinically relevant increase in the risk of depressive episodes, MDD, and stress-related disorders. These results underscore the need for integrated care approaches that include mental health screening and support as part of routine LS management. Future studies should explore whether targeted mental health interventions can mitigate psychiatric risk in this population.

## Data Availability

The raw data supporting the conclusions of this article will be made available by the authors, without undue reservation.
