# Correction: Lichen sclerosus and the association with subsequent psychiatric disorders

**DOI:** 10.3389/fmed.2026.1810216

**Published:** 2026-04-10

**Authors:** Salam Alfarsi, Andreas Recke, Katja Bieber, Diamant Thaçi, Ralf J. Ludwig, Philip Curman

**Affiliations:** 1 Lübeck Institute of Experimental Dermatology, University of Lübeck, Lübeck, Germany; 2 Department of Dermatology, University-Hospital Schleswig-Holstein (UKSH), Lübeck, Germany; 3 Institute and Comprehensive Centre for Inflammation Medicine, University-Hospital Schleswig-Holstein, Lübeck, Germany; 4 Dermato-Venereology Clinic, Karolinska University Hospital, Stockholm, Sweden; 5 Division of Dermatology and Venereology, Department of Medicine (Solna), Karolinska Institutet, Stockholm, Sweden; 6 Department of Medical Epidemiology and Biostatistics, Karolinska Institutet, Stockholm, Sweden

**Keywords:** psychiatric disease, Lichen sclerosus, TriNetX, cohort study, mental health, depression

In the published article, there were errors in the figures. Figures 2, 3 were mislabeled, and Figure 3 was missing the 95% confidence intervals (CI) for the upper two-thirds. The correct Figures 2, 3 and their captions appear below.

**Figure 2:**
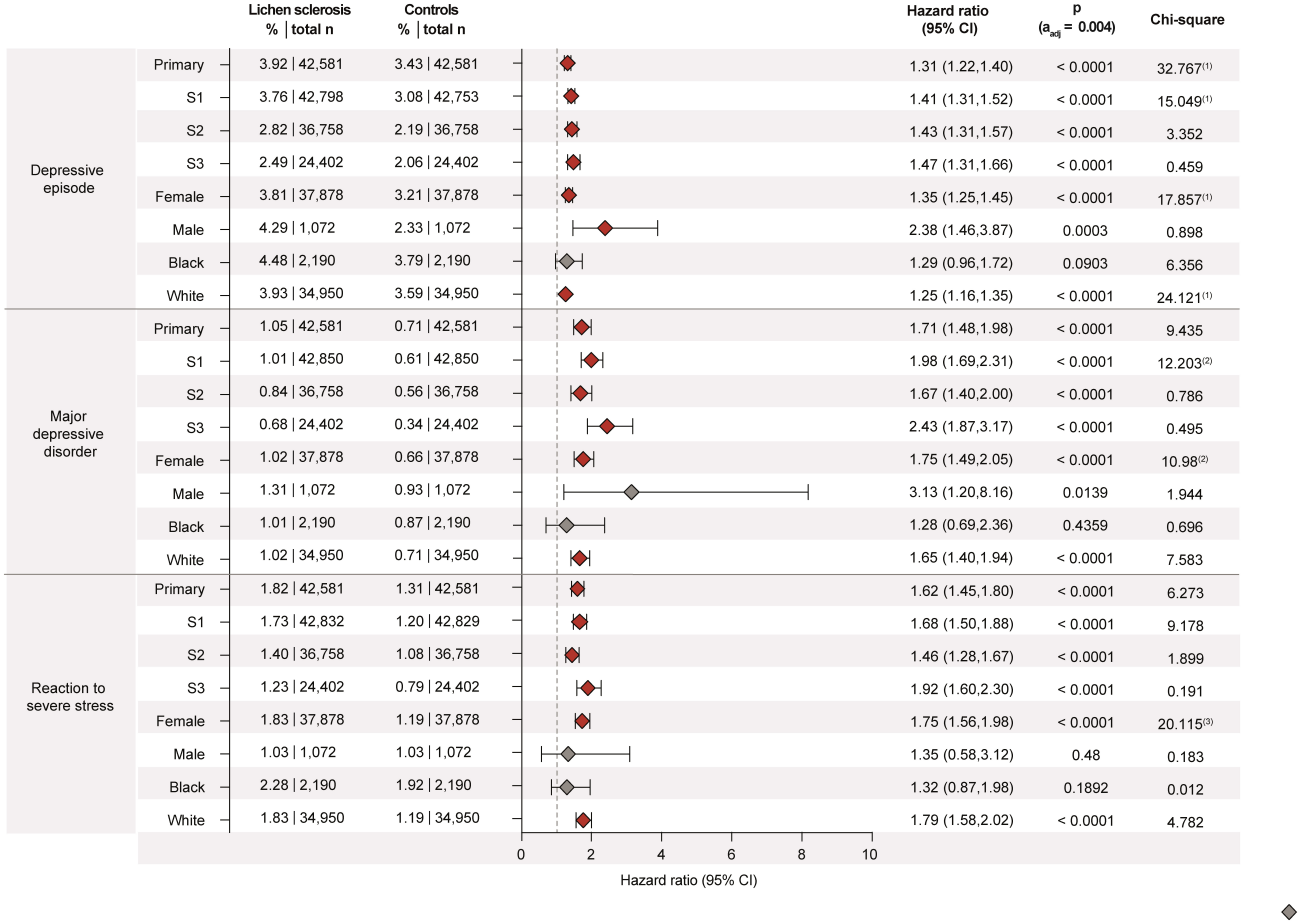
Lichen sclerosus (LS) is associated with increased risks for depression, major depressive disorder, and reaction to severe stress. Data were retrieved and analyzed using the US Collaborative Network of TriNetX. The figure displays hazard ratios (HRs) and 95% confidence intervals (CIs) for depressive episode, major depressive disorder, and reaction to severe stress in patients with LS vs. propensity-score matched non-LS controls. Risks are shown for the primary analysis, all three sensitivity analyses (S1–S3), and subgroups stratified by sex (male, female) and self-reported ethnicity (White, Black or African American). Chi-square values indicate proportionality of outcome distribution across groups. Please note that proportional hazards assumption was violated in some subgroup analyses, indicating that hazard ratios may vary over time and should be interpreted with caution.

**Figure 3:**
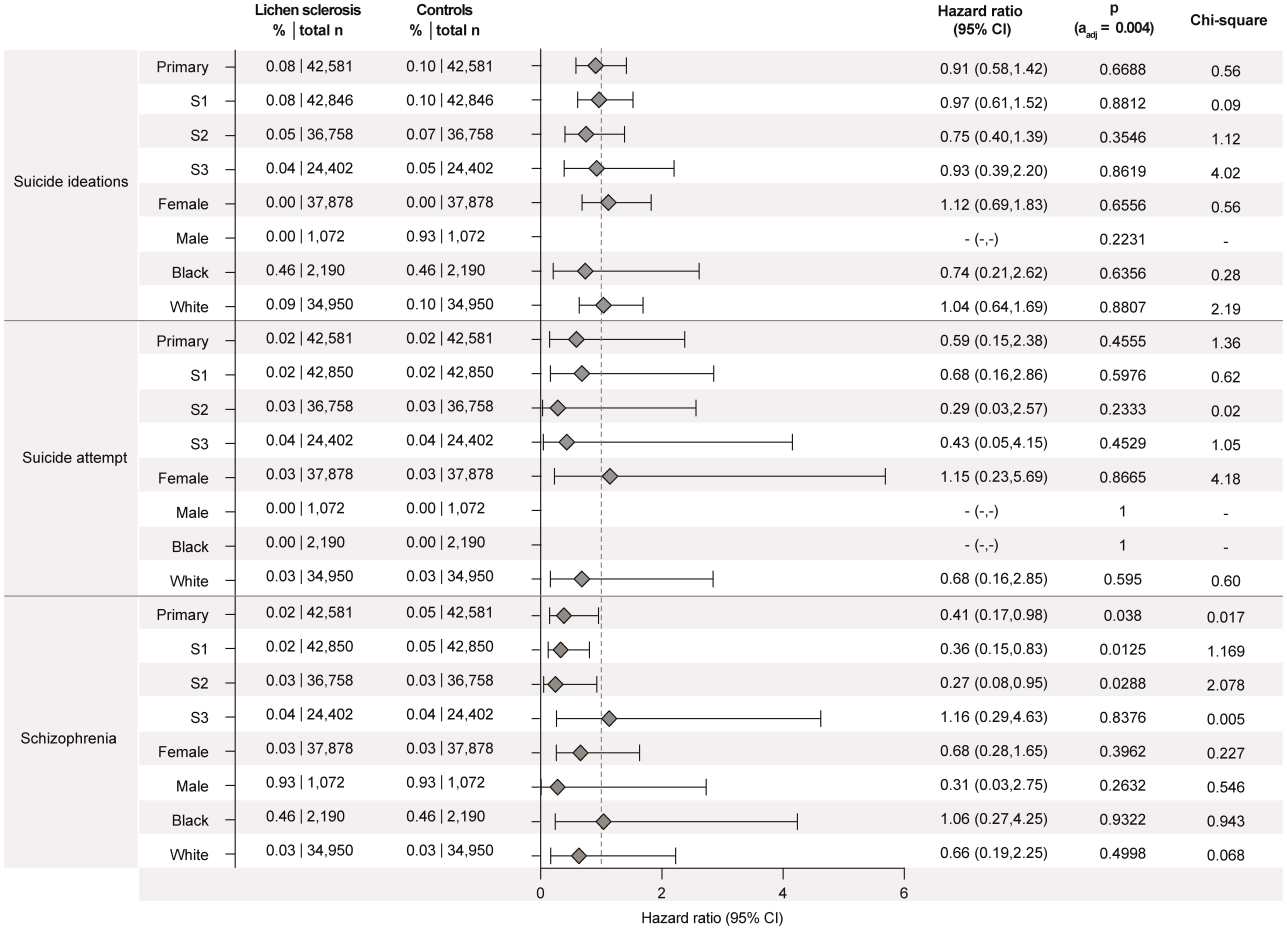
Lichen sclerosus (LS) is not associated with increased risks for suicidal ideation, suicide attempts, or schizophrenia. Data were retrieved and analyzed using the US Collaborative Network of TriNetX. The figure displays hazard ratios (HRs) and 95% confidence intervals (CIs) for suicidal ideation, suicide attempts, or schizophrenia in patients with LS vs. propensity-score matched non-LS controls. Risks are shown for the primary analysis, all three sensitivity analyses (S1–S3), and subgroups stratified by sex (male, female) and self-reported ethnicity (White, Black or African American). Chi-square values indicate proportionality of outcome distribution across groups. Please note that proportional hazards assumption was violated in some subgroup analyses, indicating that hazard ratios may vary over time and should be interpreted with caution.

The original version of this article has been updated.

