# Supplementary material for: Lichen sclerosus and the association with subsequent psychiatric disorders
Source: Front Med (Lausanne). 2025 Oct 21;12:1653347. doi: 10.3389/fmed.2025.1653347 (PMC12583063; doi:10.3389/fmed.2025.1653347)
Supplement: Supplementary file 1 [file Table_1.DOCX]

Supplementary Material

# Supplementary tables

## Supplementary table 1. Baseline characteristics before and after propensity-score matching for the lichen sclerosus cohort and controls for sensitivity analyses S2 and S3, as well as subgroups (sex and ethnicity). *SD: standard deviation; Std. diff.: standardized difference.*

| **Analysis** | **Characteristic** | **Before matching** | | | **After matching** | | |
| --- | --- | --- | --- | --- | --- | --- | --- |
|  |  | **Lichen sclerosus** | **Controls** | **Std. diff.** | **Lichen sclerosus** | **Controls** | **Std. diff.** |
| **S2** | Number of participants | 36,758 | 5,440,164 | - | 36,758 | 36,758 | - |
|  | Follow-up (days) median (interquartile range) | 734 (924) | 771 (961) | - | 734 (924) | 865 (984) | - |
|  | Age at Index (years, SD) | 62.1 ± 14.1 | 50.6 ± 18.4 | 0.7025 | 62.1 ± 14.1 | 62.2 ± 14.1 | 0.0025 |
|  | Female (%) | 94.3 | 50.6 | 1.1282 | 94.3 | 94.3 | 0.0002 |
|  | White (%) | 81.4 | 64.9 | 0.3803 | 81.4 | 81.5 | 0.0013 |
|  | Diseases of the circulatory system (I00-I99, %) | 43.7 | 38.0 | 0.1154 | 43.7 | 43.6 | 0.0015 |
|  | Diseases of the respiratory system (J00-J99, %) | 35.4 | 36.138 | 0.0151 | 35.4 | 35.4 | 0.0011 |
|  | Neoplasms (C00-D49, %) | 34.6 | 19.806 | 0.3362 | 34.6 | 34.3 | 0.0065 |
|  | Diseases of the blood and blood-forming organs and certain disorders involving the immune mechanism (D50-D89, %) | 16.0 | 14.036 | 0.0553 | 16.0 | 15.9 | 0.0038 |
|  | Diseases of the musculoskeletal system and connective tissue (M00-M99, %) | 58.3 | 46.8 | 0.2323 | 58.23 | 58.1 | 0.0036 |
|  | Endocrine, nutritional and metabolic diseases (E00-E89, %) | 56.0 | 48.3 | 0.1534 | 56.0 | 55.8 | 0.0028 |
|  | Diseases of the digestive system (K00-K95, %) | 43.8 | 33.34 | 0.2150 | 43.8 | 43.7 | 0.0019 |
|  | Overweight and obesity (E66,%) | 16.3 | 14.9 | 0.0366 | 16.3 | 16.1 | 0.0052 |
|  | Persons with potential health hazards related to socioeconomic and psychosocial circumstances (Z55-Z65, %) | 0.9 | 1.4 | 0.0423 | 0.9 | 0.9 | 0.0066 |
| **S3** | Number of participants | 24,402 | 4,974,648 |  | 24,402 | 24,402 |  |
|  | Follow-up (days) median (interquartile range) | 1,215.5 (1,223) | 1,764 (987) |  | 1,215.5 (1,223) | 1,819 (927) |  |
|  | Age at Index (years, SD) | 61 ± 13.8 | 47 ± 18 | 0.8717 | 61 ± 13.8 | 61 ± 13.8 | 0.0038 |
|  | Female (%) | 95.2 | 51.00 | 1.1512 | 95.2 | 95.2 | 0.0006 |
|  | White (%) | 82.7 | 64.3 | 0.4250 | 82.7 | 82.7 | 0.0024 |
|  | Diseases of the circulatory system (I00-I99, %) | 41.56 | 28.1 | 0.2862 | 41.56 | 41.5 | 0.0012 |
|  | Diseases of the respiratory system (J00-J99, %) | 34.9 | 29.0 | 0.1250 | 34.9 | 34.7 | 0.0024 |
|  | Neoplasms (C00-D49, %) | 34.4 | 14.8 | 0.4674 | 34.4 | 34.2 | 0.0055 |
|  | Diseases of the blood and blood-forming organs and certain disorders involving the immune mechanism (D50-D89, %) | 15.6 | 9.4 | 0.1878 | 15.6 | 15.56 | 0.0003 |
|  | Diseases of the musculoskeletal system and connective tissue (M00-M99, %) | 54.7 | 36.0 | 0.3821 | 54.7 | 54.5 | 0.0049 |
|  | Endocrine, nutritional and metabolic diseases (E00-E89, %) | 52.9 | 34.7 | 0.3737 | 52.9 | 52.7 | 0.0049 |
|  | Diseases of the digestive system (K00-K95, %) | 42.529 | 25.0 | 0.3769 | 42.5 | 42.5 | 0.0013 |
|  | Overweight and obesity (E66,%) | 14.7 | 9.23 | 0.1675 | 14.7 | 14.4 | 0.0062 |
|  | Persons with potential health hazards related to socioeconomic and psychosocial circumstances (Z55-Z65, %) | 0.9 | 1.0 | 0.0035 | 0.9 | 0.8 | 0.0173 |
| **Female** | Number of participants | 37,878 | 3,998,496 |  | 37,878 | 37,878 |  |
|  | Follow-up (days) median (interquartile range) | 1,028 (1,412) | 1,323 (1,338) |  | 1,028 (1,412) | 1,342.5 (1,309) |  |
|  | Age at Index (years, SD) | 61.1 ± 14 | 46.4 ± 18.1 | 0.9081 | 61.1 ± 14 | 61.1 ± 14 | 0.0002 |
|  | White (%) | 82.1 | 63.9 | 0.4167 | 82.1 | 82.1 | 0.0003 |
|  | Diseases of the circulatory system (I00-I99, %) | 42.4 | 26.3 | 0.3449 | 42.4 | 42.4 | 0.0006 |
|  | Diseases of the respiratory system (J00-J99, %) | 36.7 | 30.0 | 0.1440 | 36.8 | 36.7 | 0.0003 |
|  | Neoplasms (C00-D49, %) | 34.45 | 15.1 | 0.4604 | 34.5 | 34.4 | 0.0014 |
|  | Diseases of the blood and blood-forming organs and certain disorders involving the immune mechanism (D50-D89, %) | 16.8 | 11.34 | 0.1573 | 16.8 | 16.80 | 0.0004 |
|  | Diseases of the musculoskeletal system and connective tissue (M00-M99, %) | 56.6 | 36.7 | 0.4075 | 56.6 | 56.6 | 0.0001 |
|  | Endocrine, nutritional and metabolic diseases (E00-E89, %) | 54.4 | 35.45 | 0.3865 | 54.4 | 54.4 | < 0.0001 |
|  | Diseases of the digestive system (K00-K95, %) | 43.6 | 25.2 | 0.3949 | 43.6 | 43.5 | 0.0005 |
|  | Overweight and obesity (E66,%) | 15.7 | 10.3 | 0.1599 | 15.7 | 15.7 | < 0.0001 |
|  | Persons with potential health hazards related to socioeconomic and psychosocial circumstances (Z55-Z65, %) | 1.0 | 1.2 | 0.0139 | 1.0 | 1.0 | 0.0091 |
| **Male** | Number of participants | 1,072 | 2,525,163 | - | 1,072 | 1,072 | - |
|  | Follow-up (days) median (interquartile range) | 851 (1,472.5) | 1,397 (1,286) | - | 851 (1,472.5) | 1,383.5(1,272.5) | - |
|  | Age at Index (years, SD) | 56.2 ± 16.5 | 47.9 ± 17.8 | 0.4878 | 56.2 ± 16.5 | 56.3 ± 16.8 | 0.0026 |
|  | White (%) | 76.0 | 69.45 | 0.1479 | 76.0 | 74.9 | 0.0260 |
|  | Diseases of the circulatory system (I00-I99, %) | 53.6 | 33.1 | 0.4229 | 53.56 | 53.5 | 0.0019 |
|  | Diseases of the respiratory system (J00-J99, %) | 41.1 | 29.2 | 0.2508 | 41.01 | 40.2 | 0.0171 |
|  | Neoplasms (C00-D49, %) | 37.2 | 13.9 | 0.5562 | 37.2 | 37.2 | < 0.0001 |
|  | Diseases of the blood and blood-forming organs and certain disorders involving the immune mechanism (D50-D89, %) | 19.4 | 9.149 | 0.2963 | 19.4 | 20.14 | 0.0187 |
|  | Diseases of the musculoskeletal system and connective tissue (M00-M99, %) | 57.6 | 37.7 | 0.4055 | 57.6 | 59.1 | 0.0303 |
|  | Endocrine, nutritional and metabolic diseases (E00-E89, %) | 58.7 | 35.7 | 0.4740 | 58.7 | 59.0 | 0.0057 |
|  | Diseases of the digestive system (K00-K95, %) | 51.7 | 27.4 | 0.5131 | 51.7 | 51.0 | 0.0131 |
|  | Overweight and obesity (E66,%) | 25.2 | 9.2 | 0.4335 | 25.2 | 25.4 | 0.0043 |
|  | Persons with potential health hazards related to socioeconomic and psychosocial circumstances (Z55-Z65, %) | 2.1 | 1.02 | 0.0835 | 2.1 | 2.8 | 0.0485 |
| **Black or African American** | Number of participants | 2,190 | 1,085,544 |  | 2,190 | 2,190 |  |
|  | Follow-up (days) median (interquartile range) | 1,042 (1,436) | 1,188 (1,397) |  | 1,042 (1,436) | 1,210 (1,347) |  |
|  | Age at Index (years, SD) | 58.5 ± 14.6 | 45.3 ± 17.7 | 0.8176 | 58.5 ± 14.6 | 58.8± 14.6 | 0.0175 |
|  | Female (%) | 94.5 | 59.5 | 0.9164 | 94.5 | 94.4 | 0.0040 |
|  | White (%) | 0 | 0 | - | 0 | 0 | - |
|  | Diseases of the circulatory system (I00-I99, %) | 55.5 | 35.1 | 0.4189 | 55.5 | 56.5 | 0.0202 |
|  | Diseases of the respiratory system (J00-J99, %) | 44.6 | 30.6 | 0.2923 | 44.6 | 44.5 | 0.0018 |
|  | Neoplasms (C00-D49, %) | 32.8 | 12.34 | 0.5051 | 32.8 | 32.6 | 0.0058 |
|  | Diseases of the blood and blood-forming organs and certain disorders involving the immune mechanism (D50-D89, %) | 26.0 | 14.7 | 0.2831 | 26.0 | 26.3 | 0.0052 |
|  | Diseases of the musculoskeletal system and connective tissue (M00-M99, %) | 57.6 | 37.7 | 0.4055 | 57.6 | 59.01 | 0.0303 |
|  | Endocrine, nutritional and metabolic diseases (E00-E89, %) | 58.7 | 35.7 | 0.4740 | 58.7 | 59.0 | 0.0057 |
|  | Diseases of the digestive system (K00-K95, %) | 51.7 | 27.4 | 0.5131 | 51.7 | 51.0 | 0.0131 |
|  | Overweight and obesity (E66,%) | 25.2 | 9.2 | 0.4335 | 25.2 | 25.4 | 0.0043 |
| **White** | Number of participants | 34,950 | 4,891,667 | - | 34,950 | 34,950 | - |
|  | Follow-up (days) median (interquartile range) | 1,044 (1,405) | 1,393 (1,317) | - | 1,044 (1,405) | 1,422 (1,285) | - |
|  | Age at Index (years, SD) | 61.4 ± 13.8 | 48.3 ± 18.1 | 0.8168 | 61.4 ± 13.8 | 61.4 ± 13.8 | 0.0004 |
|  | Female (%) | 96.1 | 51.7 | 1.1695 | 96.1 | 96.1 | 0.0006 |
|  | Diseases of the circulatory system (I00-I99, %) | 43.4 | 30.1 | 0.2775 | 43.4 | 43.3 | 0.0014 |
|  | Diseases of the respiratory system (J00-J99, %) | 37.3 | 30.7 | 0.1381 | 37.23 | 37.2 | 0.0008 |
|  | Neoplasms (C00-D49, %) | 36.3 | 16.5 | 0.4617 | 36.3 | 36.3 | 0.0004 |
|  | Diseases of the blood and blood-forming organs and certain disorders involving the immune mechanism (D50-D89, %) | 16.3 | 9.9 | 0.1907 | 16.3 | 16.3 | 0.0005 |
|  | Diseases of the musculoskeletal system and connective tissue (M00-M99, %) | 57.6 | 38.56 | 0.3869 | 57.6 | 57.5 | 0.0004 |
|  | Endocrine, nutritional and metabolic diseases (E00-E89, %) | 54.9 | 36.4 | 0.3766 | 54.8 | 54.8 | 0.0010 |
|  | Diseases of the digestive system (K00-K95, %) | 44.7 | 27.2 | 0.3710 | 44.7 | 44.7 | 0.0001 |
|  | Overweight and obesity (E66,%) | 15.1 | 9.4 | 0.1732 | 15.1 | 15.0 | 0.0031 |
|  | Persons with potential health hazards related to socioeconomic and psychosocial circumstances (Z55-Z65, %) | 1.0 | 1.0 | 0.0039 | 1.0 | 1.0 | 0.0052 |
